# Untargeted Metabolomic Analysis of Human Plasma Indicates Differentially Affected Polyamine and L-Arginine Metabolism in Mild Cognitive Impairment Subjects Converting to Alzheimer’s Disease

**DOI:** 10.1371/journal.pone.0119452

**Published:** 2015-03-24

**Authors:** Stewart F. Graham, Olivier P. Chevallier, Christopher T. Elliott, Christian Hölscher, Janet Johnston, Bernadette McGuinness, Patrick G. Kehoe, Anthony Peter Passmore, Brian D. Green

**Affiliations:** 1 Advanced Asset Technology Centre, Institute for Global Food Security, Queen’s University Belfast, Stranmillis Road, Belfast, BT9 5AG, United Kingdom; 2 William Beaumont Research Institute, 3811 W. 13 Mile Road, Royal Oak, Michigan 48073, United States of America; 3 Division of Biomedical Sciences and Life Sciences, Lancaster University, Lancaster, LA1 4YG, United Kingdom; 4 Ageing Group, Centre for Public Health, Queen's University Belfast, Belfast, BT12 6BA, United Kingdom; 5 Dementia Research Group, Institute of Clinical Neurosciences, School of Clinical Sciences, University of Bristol, Frenchay Hospital, Bristol, BS16 1LE, United Kingdom; Pacific Northwest National Laboratory, UNITED STATES

## Abstract

This study combined high resolution mass spectrometry (HRMS), advanced chemometrics and pathway enrichment analysis to analyse the blood metabolome of patients attending the memory clinic: cases of mild cognitive impairment (MCI; *n = 16*), cases of MCI who upon subsequent follow-up developed Alzheimer’s disease (MCI_AD; *n = 19*), and healthy age-matched controls (Ctrl; *n = 37*). Plasma was extracted in acetonitrile and applied to an Acquity UPLC HILIC (1.7μm x 2.1 x 100 mm) column coupled to a Xevo G2 QTof mass spectrometer using a previously optimised method. Data comprising 6751 spectral features were used to build an OPLS-DA statistical model capable of accurately distinguishing Ctrl, MCI and MCI_AD. The model accurately distinguished (R2 = 99.1%; Q2 = 97%) those MCI patients who later went on to develop AD. S-plots were used to shortlist ions of interest which were responsible for explaining the maximum amount of variation between patient groups. Metabolite database searching and pathway enrichment analysis indicated disturbances in 22 biochemical pathways, and excitingly it discovered two interlinked areas of metabolism (polyamine metabolism and L-Arginine metabolism) were differentially disrupted in this well-defined clinical cohort. The optimised untargeted HRMS methods described herein not only demonstrate that it is possible to distinguish these pathologies in human blood but also that MCI patients ‘at risk’ from AD could be predicted up to 2 years earlier than conventional clinical diagnosis. Blood-based metabolite profiling of plasma from memory clinic patients is a novel and feasible approach in improving MCI and AD diagnosis and, refining clinical trials through better patient stratification.

## Introduction

Alzheimer’s disease (AD) is a progressive neurodegenerative disorder for which there is no cure and few reliable diagnostic biomarkers [1]. AD is the most common form of dementia and diagnosis rates are continually rising, driven partly by the increasing age profile of Western societies. At present AD therapies are initiated only after diagnosis and their modest efficacy is due to them only treating symptoms due to chemical imbalance that only partially compensates for the considerable irreversible brain damage that has already occurred by the time of diagnosis. Therefore, valid and reliable biomarkers capable of detecting early AD pathogenesis are vital for patients, clinicians, researchers, and care commissioners. Early recognition, assessment and diagnosis of AD are key priorities in all national dementia strategies. Current research strategies around biomarkers for early diagnosis focus on measurement of levels of amyloid beta(Aβ), the Aβ (1–40)/(1–42) ratio, levels of phosphorylated tau [1], and Aβ/tau ratio in cerebrospinal fluid (CSF). Increasingly, imaging techniques are also being widely researched and increasingly used in clinical settings whereby levels of brain atrophy by MRI are being used to aid with diagnosis, while the measurement of plaque load in PET imaging studies is still very much a research application [2]. The predictive value of these techniques remains limited so the investigation of novel sensitive and specific methods such as high resolution mass spectrometry (HRMS) metabolomics is still warranted [3].

Mild cognitive impairment (MCI) is considered to be a transitional phase between normal aging and AD (Mild cognitive impairment as a diagnostic entity [4]). Patients with MCI have cognitive impairment, primarily in their memory functions but their daily living activities are relatively unaffected, and as a condition, MCI does not fulfil the criteria of AD or any other form of dementia. MCI is heterogeneous in nature with several potential outcomes, ranging from increased risk of developing AD to restoration of normal cognition [5–7]. There is currently no clinical method of accurately determining which MCI subjects will later progress to AD. Therefore, leading-edge research studies focus on identifying features which differentiate stable MCI subjects, MCI subjects later converting to AD, and healthy age-matched control subjects.

Metabolomics is a powerful tool for characterising complex disease phenotypes where there are both genetic and environmental components. It is a discipline dedicated to the global study of small molecules metabolites in cells, tissues and biofluids [8]. It involves the comprehensive, simultaneous and systematic profiling of numerous metabolite concentrations and their fluctuations in response to disease, drugs, diet or lifestyle [8]. Metabolomics datasets are frequently large, complex and difficult to interpret but the combination of several cutting-edge bio-informatic techniques (i.e. multivariate statistics, metabolite database searching, and pathway enrichment analysis) makes it possible to visualise and biologically interpret metabolite data on a system level.

‘Targeted’ and ‘untargeted’ metabolomics techniques have been used to profile CSF samples from dementia patients. Recently, a targeted LC-electrochemical array approach measured the concentrations of 71 known and 24 unknown metabolites in CSF [9]. The model generated classified control, MCI and AD subjects with 83.1% predictive accuracy, and this approach meant that it was straightforward to quickly identify 4 metabolic pathways that were impacted: tryptophan, tyrosine, methionine and purine metabolism [9]. Untargeted methods on-the-other-hand are less prone to bias because they do not predefine which metabolites should be measured [10], however, this has the disadvantage that it can be slow and difficult to identify metabolites or pathways. For example, CSF profiling (control, MCI and AD subjects) recently generated models with >95% specificity and sensitivity following cross-validation[11]. However, from 11,549 spectral features initially analysed just 17 ‘metabolites’ were shortlisted of which 6 were positively identified [11]. The information gained can be valuable but such an approach limits the number of potential findings from what are extremely large datasets. Encouragingly new bioinformatics tools can improve ‘untargeted’ metabolomics by combining automated database searching with pathway enrichment analysis. This assigns putative metabolite identities on the basis of isotope similarity and mass error, and then identifies coordinated changes along known biochemical pathways.

The aims of this investigation were to: (1) develop and optimise a HRMS metabolomics method capable of capturing the maximum number of spectral features in human plasma; (2) use this method to profile the plasma of patients attending a memory clinic, including: cases of mild cognitive impairment (MCI), cases of MCI who subsequently went on to develop AD (MCI_AD), and healthy age-matched controls (Ctrl); (3) develop and validate multivariate models with high specificity and sensitivity to accurately differentiate patient groups; and (4) employ pathway enrichment analysis to identify the areas of metabolism which are affected in each clinical state.

## Materials and Methods

### Ethics Statement

Appropriate research ethical approval at Queen’s University Belfast was sought and obtained. Written informed consent was obtained from all participants.

### Study design and participants

Patients (n = 139) and Healthy Age-Matched Controls (n = 98) were recruited from the Belfast City Hospital memory clinic. Of this sample cohort we selected n = 37 control samples, n = 16 MCI subjects and n = 19 MCI_AD subjects, matching age as closely as possible and splitting each group equally into male and female subgroups. Demographic characteristics are summarised in S1 Table. Patients presented with subjective memory problems usually but were functionally independent and scored ≥24/30 on the MMSE [12] and 82–88/100 on the Addenbrookes Cognitive Examination-Revised [13]. Patients were diagnosed with MCI according to criteria developed by an international working group on MCI [14]. Controls were recruited from groups of volunteers that have previously assisted with studies of this type or were spouses of patients. They had no cognitive complaints subjectively or objectively and were physically and mentally healthy.

The neuropsychological evaluation comprised speed and attention, learning and episodic memory, visuospatial function, language and executive function as recommended by the American Academy of Neurology (AAN). Within each cognitive domain several aspects of function were assessed in order to obtain as complete a picture as possible. In order to differentiate cognitive domains a cut off for each test was set at 1.5 standard deviations below the control mean. An impaired result on at least one test in each cognitive domain was required to be considered impaired in the domain. MCI patients were then assigned to groups: amnestic single domain, amnestic multidomain, nonamnestic single domain and nonamnestic multidomain. The resultant cognitive classification is illustrated in S2 Table. The neurological assessment was repeated at year 1 and 2 as applicable. Controls were assessed once only.

The diagnosis of AD was made using NINCDS-ADRDA criteria [15]. Most of the MCI_AD patients belonged to the amnestic multidomain group initially as per previous neuropsychological studies of this type [16–18].

Plasma was collected in EDTA tubes from individuals using standard venepuncture procedures. It was immediately centrifuged and stored in aliquots at -80°C until the date of analysis.

### Preparation of plasma extracts

Based on the results of previous studies the pool of polar metabolites were hypothesised to be the best for identifying potential biomarker candidates of AD [19]. Samples were prepared by eluting plasma through an Ostro Plate (Waters, Ireland) in accordance with the manufacturer’s instructions. The Ostro plate provided a high-throughput reproducible method of removing proteins and phospholipids with minimal sample preparation. Briefly, each test sample (n = 80) a 100 μl aliquot of plasma was transferred to its respective well on the 96-well Ostro plate. To each well 400 μl of acetonitrile containing 1% formic acid was added and mixed thoroughly by aspirating five times. The samples were subsequently filtered under vacuum for five minutes into a clean collection plate (Waters, Manchester) and were immediately transferred to the UPLC-QTof-MS for analysis.

### LC-QTof-MS Analysis

An exhaustive process of optimisation was undertaken which assessed various column chemistries, extraction procedures, solvent gradients, modes of acquisition and mass spectrometer settings. The following protocol was found to be optimal for the analysis of polar plasma metabolites capturing the maximal number of spectral features in the most reproducible manner. All solvents (water, acetonitrile, formic acid, ammonia solution 25%) were purchased from Sigma-Aldrich (Dorset, UK) and were LC-MS grade or equivalent. Chromatography was performed on a Waters Acquity UPLC I-Class system(Milford, MA, USA), equipped with column oven, coupled to a Waters Xevo G2 QTof mass spectrometer (Manchester, UK) equipped with an electrospray ionisation source operating in positive mode with lock-spray interface for real time accurate mass correction. The source temperature was 120°C with a cone gas flow of 5 L/h, a desolvation temperature of 350°C, and a desolvation gas flow of 600 L/h. The capillary voltage was set at 0.3 kV with a cone voltage of 20 V.

A lock-mass solution of Leucine Enkephalin (2 ng.μL-1) in acetonitrile/water containing 0.1% formic acid (50:50, v/v) was continuously infused into the MS via the lock-spray at a flow rate of 5 μl.min-1. Leucine Enkephalin is a commonly used peptide, which has been studied in detail and is commonly used as a standard or reference compound to calibrate mass spectrometers during analysis. This ensures accurate masses (±2 ppm) are obtained during every analytical run. Mass spectra data were acquired in centroid mode using MSE function (low energy: 4eV; high energy: ramp from 20 to 35 eV) over the range m/z 50–1200 range with a scan time of 0.1s.

A 1.0 μL aliquot of extracted plasma sample was injected onto an Acquity UPLC BEH HILIC column (2.1 x 100 mm, 1.7 μm, Waters, Milford, MA, USA). The main principle of HILIC (Hydrophilic interaction chromatography) separation is based on a compounds polarity and degree of solvation. The more polar compounds are separated by their stronger interaction with the stationary aqueous layer than the less polar compounds, therefore resulting in a stronger retention on the analytical column [19]. The column oven was set at 45°C, and the sample manager temperature was 6°C. The gradient elution buffers were A (5 mM ammonium formate) and B (acetonitrile containing 0.025% formic acid), and the flow rate was 0.6 mL.min-1. The elution gradient (A:B, v/v) was as follows: an isocratic period of 2 min at 5:95 followed by a linear gradient from 5:95 to 30:70 over 8 min then a linear change from 30:70 to 90:10 over 1 min. After a 1 min period at 90:10, a linear gradient was applied over 0.5 min to return to the initial composition 5:95 which was held for 3.5 min before the next injection.

Prior to the analysis 10 pooled conditioning samples were injected. To determine the chromatographic reproducibility of retention times and peak intensities, pooled samples were injected at intervals every 10 samples throughout the entire experiment [19].

### Data analysis

Raw data from the spectral analysis of plasma extracts was initially processed using Transomics (version 1.0; Waters Corporation, Milford, MA) software and was normalised to the total spectral intensity. The simple, automatic workflow took less than 48 hours to retention-time align all runs against a pool sample run, peak pick, deconvolute adduct ions and calculate the ion abundance of 6751 ions of interest. All detected ions were selected against the Progenesis Metascope “Biomolecules” database [20]. 1486 compound identifications were returned and automatically linked to the compounds. Putative ID’s were accepted on the basis that isotope similarity and mass error (in that order) with only one ID assigned per ion of interest (See S1 Dataset). Following this two separate data analysis approaches were undertaken: firstly multivariate data analysis was conducted to assess the suitability of HRMS profiling to accurately distinguish Ctrl, MCI and MCI_AD samples, and secondly pathway enrichment analysis was conducted to implicate potential areas of metabolism which are affected.

### Multivariate Data Analysis

The analysed spectral data was exported to Simca 13 (Umetrics, Umea, Sweden) for multivariate analysis. Prior to any in-depth data analysis, data quality was assessed in terms of reproducibility by an approach adopted by ourselves and other leading metabolomics researchers [19,21]. Clustering of the pooled samples was assessed using principal component analysis (PCA) to reveal if platform stability had been achieved. Tight clustering of pooled sample data indicated that data acquisition was highly reproducible [19]. Data were then mean centered; Pareto scaled and grouped into Controls, MCI and MCI_AD prior to analysis using orthogonal projection to latent structures-discriminant analysis (OPLS-DA) [19]. Pareto scaling was used since it augments the representation of the low concentration metabolites by dividing each variable by the square root of the standard deviation of the variable, without increasing the noise contribution to the model [22]. R2 (cumulative), Q2 (cumulative) and Root Mean Squared Error of cross validation (RMSECV) were used to determine the validity of the model. R2 (cum) indicates the variation described by all components in the model and Q2 is a measure of how accurately the model can predict class membership. Essentially it validates the statistical model by leaving 1/7^th^ of the data out of the model and then predicting their class membership [23].

Groups were compared two at a time i.e Controls vs MCI, Controls vs MCI_AD, MCI vs MCI_AD. The ions of interest (identified by the s-plot) to be at different levels between the two sample groups were analysed using a one tailed homoscedastic Students T-test (Excel 2013 (Microsoft, Redmont, MA, USA).

### Pathway Enrichment Analysis

Following multivariate analysis, the normalised data was filtered in Excel 2013 (Microsoft, Redmont, MA, USA). Firstly, ions of interest containing >20% “zero” values across all samples were excluded in order to remove spurious and inaccurate measurements Secondly, data without assigned putative ID’s were removed, and thirdly those with a p-value >0.05 were removed as calculated from the Student’s T-test (as previously described).

The filtered and putatively identified metabolites were analysed for pathway enrichment using Metacore (Thompson Reuters, Genego, Saint Joseph, MI) with metabolite names, p-values and exact molecular masses uploaded for analysis. The—log (p-value) produced by Metacore indicates the enhancement of certain metabolites in a biochemical pathway [24]. Displayed pathways were selected on the basis of their—log (p-value) and false discovery rate as produced by Metacore (both with values <0.05). Ratios indicate the number of significantly altered metabolites in the pathway against the total number of metabolites in that pathway.

## Results

### Metabolomic profiling of plasma using HRMS

**Fig 1:**
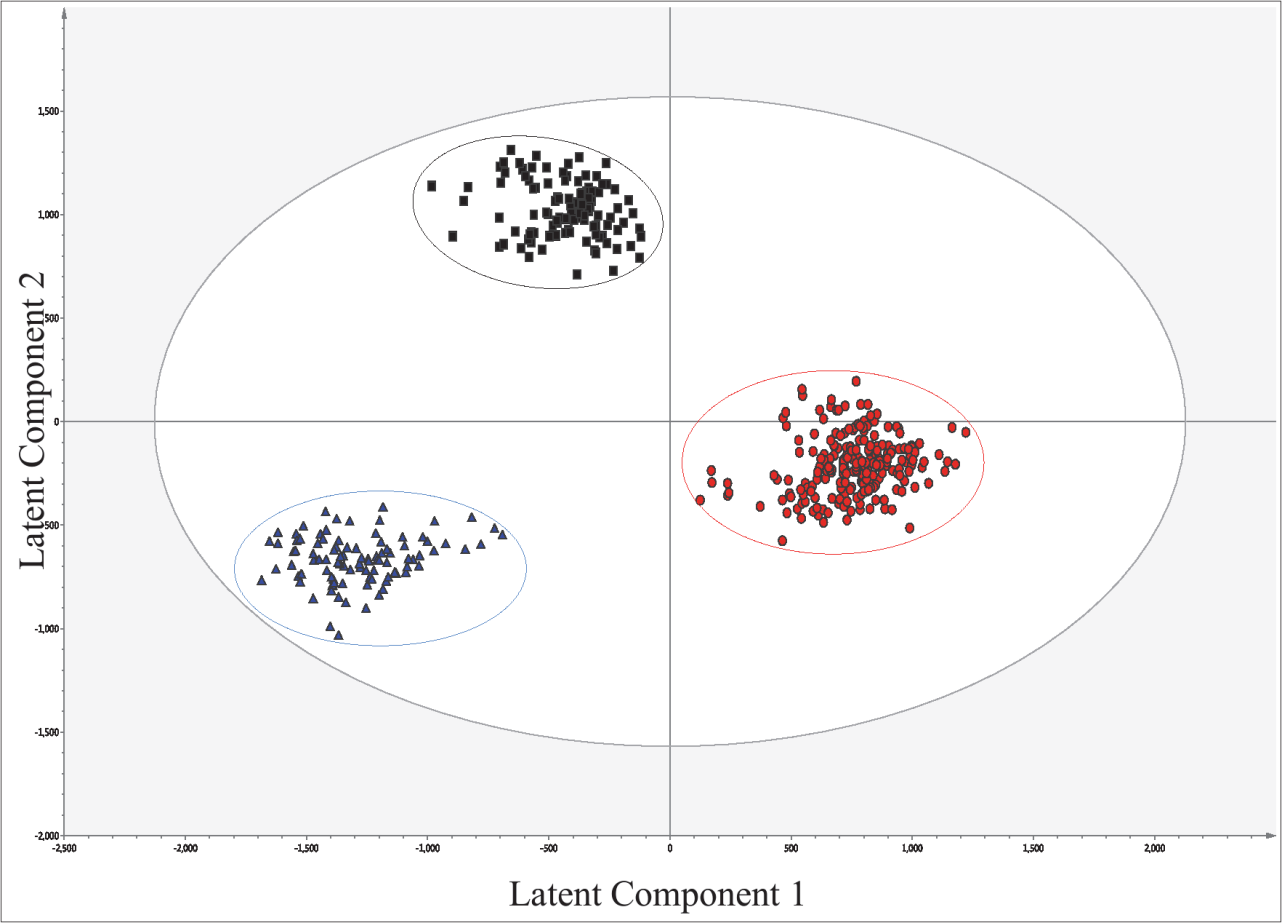
Multivariate model generated from HRMS analysis of human plasma. The OPLS-DA scores plot shows Controls (red circles), MCI (black squares) and MCI_AD (blue triangles). R2 = 95.6% and Q2 = 91.6%. R2 (cumm) indicates the variation described by all components in the model and Q2 is a measure of how accurately the model can predict class membership. Essentially it validates the statistical model by leaving 1/7th of the data out of the model and then predicting their class membership [24].

**Fig 2:**
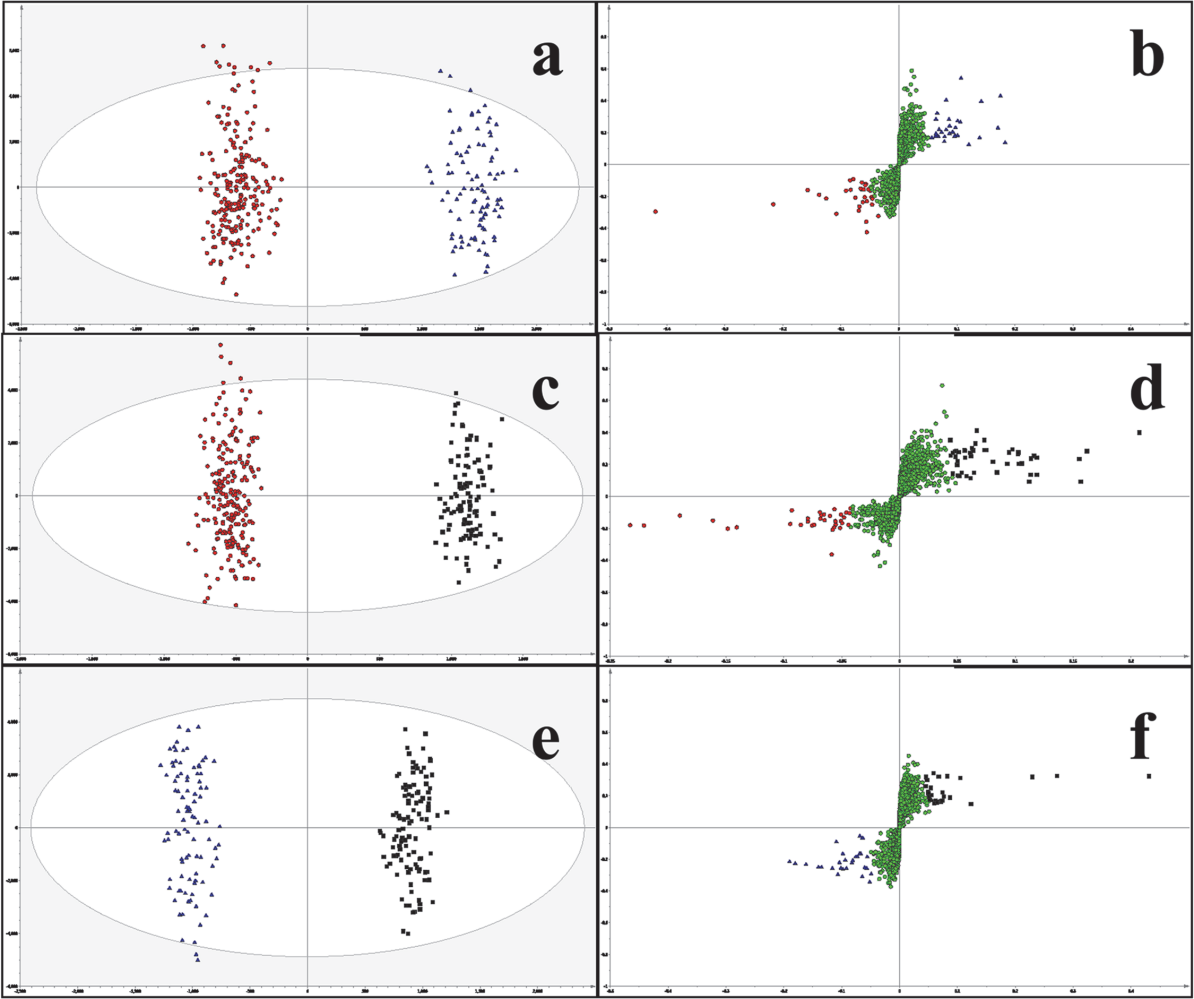
Multivariate models comparing groups and s-plots indicating the ‘ions of interest’. Groups were compared in pairs with Controls (red circles), MCI (black squares) and MCI_AD (blue triangles) indicated on OPLS-DA scores plots (Fig. 2A, 2C and 2E). (a) Ctrl vs. MCI; R2 = 97.6%; Q2 = 95.3%; (c) Ctrl vs. MCI_AD; R2 = 98.4%; Q2 = 95.7%. (f) MCI vs. MCI_AD; R2 = 98.7%; Q2 = 95.8%. Corresponding s-plots (Fig. 2B, 2D and F) highlight which ions are up or down regulated in their respective group as represented by the colour and shape of the variables.

Fig. 1 displays the multivariate analysis results when all three groups where analysed using OPLS-DA. This model and the subsequent models (Fig. 2A, 2C and 2E) were produced using 6,751 spectral features obtained by HRMS analysis. This approach clearly distinguished Ctrl, MCI and MCI_AD groups. For this model (Fig. 1) two latent components and thirteen orthogonal components were calculated with resulting R2 = 95.6%, Q2 = 91.6% and a root mean squared error of cross validation (RMSECV) of 12.2%. Fig. 2A displays the OPLS-DA scores plot for control vs. MCI accompanied by its respective s-plot in Fig. 2B. This model was created using one latent component and eight orthogonal components producing R2 = 97.6%, Q2 = 95.3% and RMSECV = 9.97%. The s-plot highlighted 33 metabolites to be at higher relative abundances in Ctrl (red-circles) and 30 to be at higher levels in MCI plasma (blue triangles) sample. However when the raw data was analysed closely only 30 of the features identified to be at higher levels in controls were considered true peaks/ions of interest and 18 out of the 30 for the MCI samples.

Fig. 2C displays the scores plot control vs. MCI_AD plasma samples complemented with its respective s-plot in Fig. 2D. This discriminant model was generated using one latent component and nine orthogonal components producing R2 = 98.4%, Q2 = 95.7% and RMSECV = 9.77%. As previously described the respective s-plot in Fig. 1E highlights features that are at significantly higher or lower levels in both controls and diseased samples. In this model the s-plots pinpoint 33 ions of interest to be higher in both Ctrl and MCI_AD samples. However when the raw data was analysed closely only 25 were found to be at higher abundances in Ctrl and 32 to be at higher levels in MCI_AD.

Fig. 2E presents the scores plots for the OPLS-DA model for MCI vs. MCI_AD accompanied by its respective s-plot in Fig. 2F. The model was created using one latent component and eight orthogonal components with R2 = 98.7%, Q2 = 95.8% and RMSECV = 10.26%. The s-plot (Fig. 2E) highlights 29 features to be at higher abundances in plasma samples taken from MCI patients whilst 15 are at higher levels in plasma samples harvested from MCI_AD patients. When the raw data was analysed 13 were found to be at higher abundance in MCI plasma samples and 13 were found to be at higher levels in plasma samples taken from MCI_AD patients. Thus, by this approach a list of 131 ions was compiled that after the removal of duplicates identified 90 features important in the 3 models distinguishing patient groups. We further confirmed the validity of our models by examining these 90 individual spectral features referred to as ‘ions of interest’. When the intensities of these ions were compared by univariate analysis 98% (i.e. 88 out of 90) were statistically significant (p-values 0.05 to 5.70e^-52^; Student’s t-test.). Therefore multivariate models discriminated between clinical cases on the basis of genuine changes in the levels of plasma ions/metabolites.

### Pathway Enrichment Analysis

**Fig 3:**
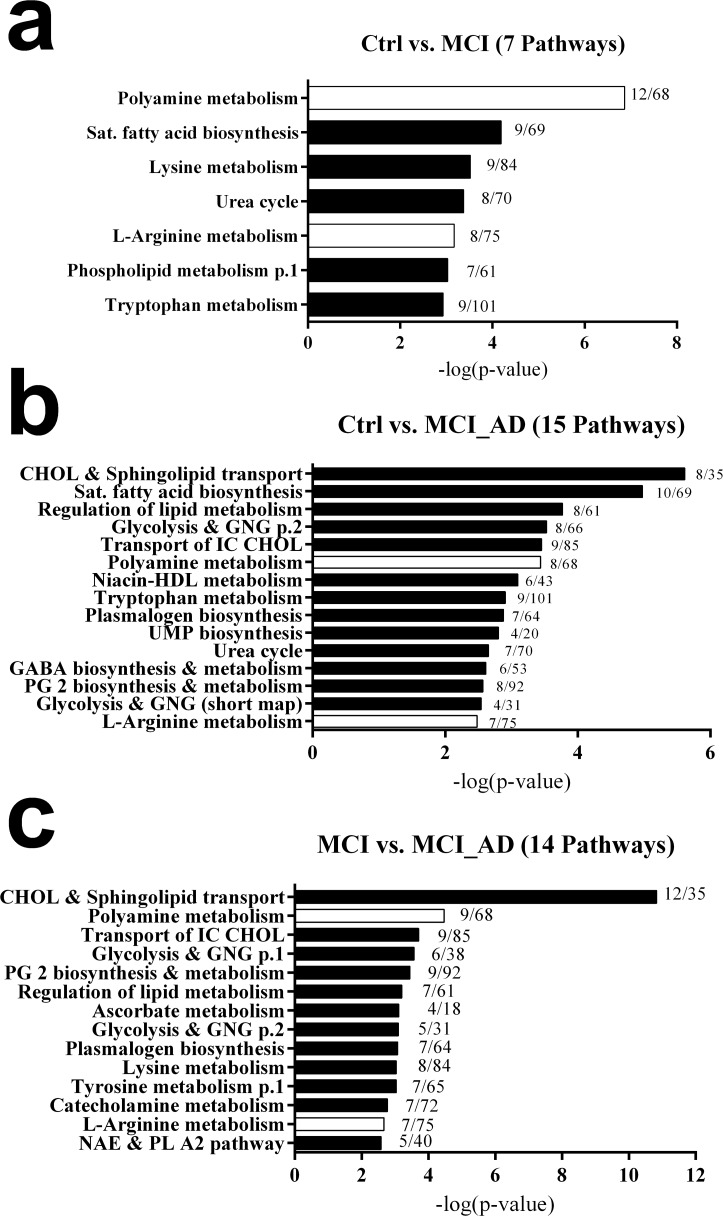
Pathway Enrichment Analysis. Altered metabolic pathways and process networks in plasma of (a) Ctrl vs. MCI, (b) Ctrl vs. MCI_AD and (c) MCI vs. MCI_AD subjects were uncovered using Metacore. The significance of the pathways was evaluated using P values and a false discovery rate of <0.05. Bars in white are pathways implicated across all three comparisons. Ratios are the number of significantly altered metabolites in the pathway against the total number of metabolites in that pathway.

**Fig 4:**
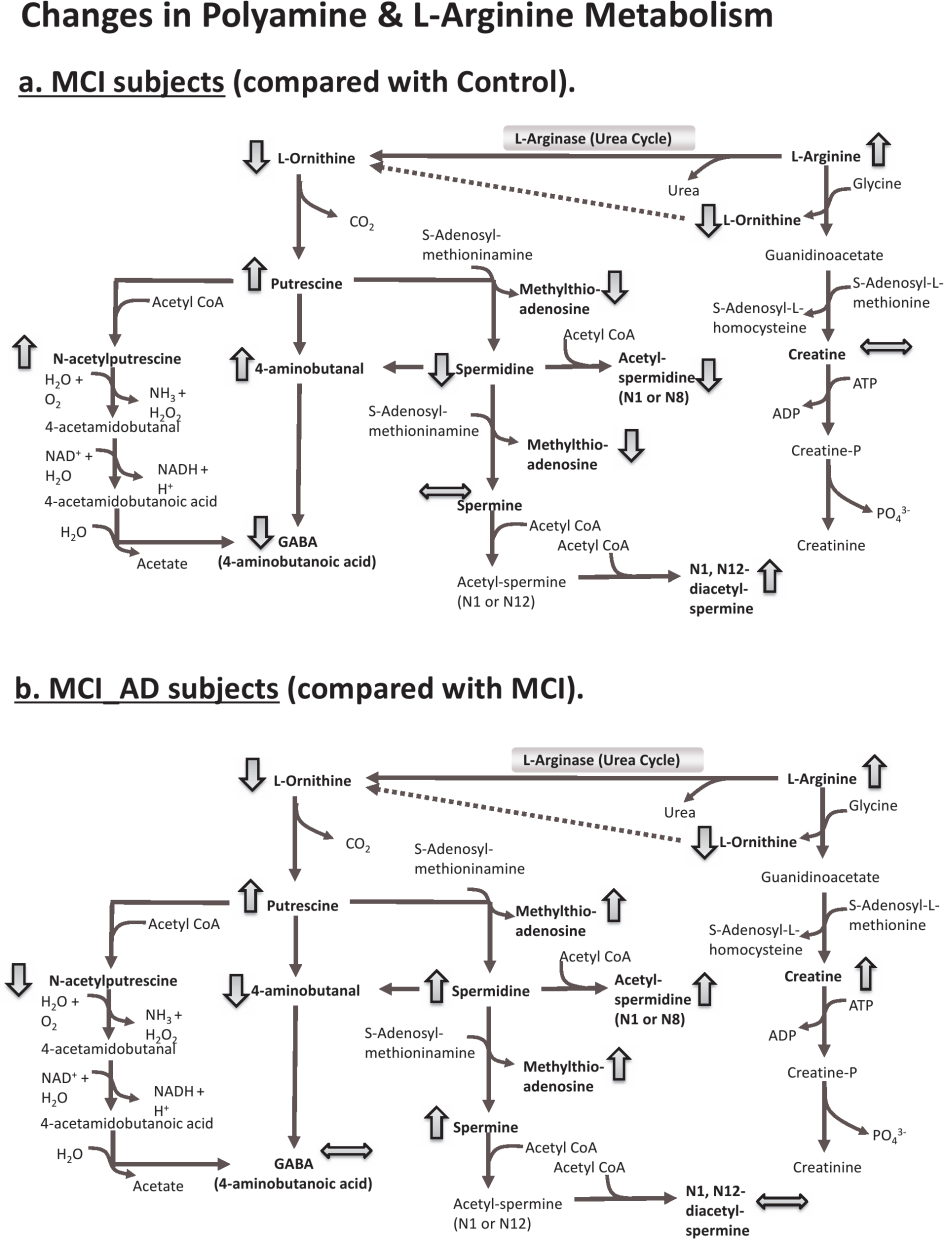
Changes in Polyamine and L-Arginine Metabolism. Pathway enrichment data indicates that *Polyamine metabolism* and *L-arginine metabolism* are differentially affected in stable MCI subjects (a.) and MCI subjects converting to AD subjects (b.). Conversion to AD (b.) preferentially channels putrescine towards the production of spermidines and spermines, whereas in stable MCI (a.) the fate of putrescine is the formation of N-acetylputrescine and 4-aminobutanal. Decline in the production of the neurotransmitter GABA occurs in stable MCI (a.) and is not further affected by conversion to AD. GABA decline is associated with a build-up of its precursor metabolites, N-acetylputrescine and 4-aminobutanal. Arrows indicate increases (↑), decreases (↓) or unchanged (↔) levels of metabolites.

Fig. 3 summarises the results of pathway enrichment analysis conducted using Metacore (Thompson Reuters, Genego, St. Joseph, MI). The importance of implicated biochemical pathways is indicated by—log (P-value) and ratios depict the number of affected metabolites to the total number of metabolites in the pathway. For controls versus MCI a total of 263 (159 elevated in MCI; 104 elevated in control) statistically significant (p<0.05) putatively identified metabolites implicated 7 biochemical pathways (Fig. 3A). For control versus MCI_AD a total of 162 (102 elevated in MCI_AD; 60 elevated in control) statistically significant (p<0.05) putatively identified metabolites implicated 15 biochemical pathways (Fig. 3B). For MCI versus MCI_AD 183 (96 elevated in MCI_AD; 87 elevated in MCI) statistically significant (P<0.05) putatively identified metabolites implicated 14 biochemical pathways. When all implicated pathways were considered together (see Venn diagram in S1 Fig.) relatively few were commonly affected across all groups. Of the indicated pathways just 2 (Polyamine metabolism and L-arginine metabolism) were common between to control, MCI and MCI_AD subjects. Interestingly these pathways are overlapping areas of metabolism sharing some metabolite intermediates. Table 1 lists the individual metabolites affected within the pathways with percentage changes and p-values for each comparison. Fig. 4 illustrates the biochemical pathways these metabolites are involved in and how they are affected in MCI and MCI_AD.

**Table 1:** Polyamine and L-Arginine metabolites identified by pathway analysis using Metacore. The table shows the % change in levels alongside p values of putative metabolites. N/A-Not affected; NS-Not Significant.

	Ctrl vs. MCI		Ctrl vs. MCI_AD		MCI vs. MCI_AD	
	% Change in MCI	P-value	% Change in MCI_AD	P-value	% Change in MCI_AD	P-value
**4-aminobutanal**	+9.97	0.027	-8.98	0.039	-17.23	0.00049
**Creatine**	N/A	NS	+22.49	0.0015	+29.07	0.00036
**GABA**	-87.00	0.04	-86.99	0.039	N/A	NS
**L-arginine**	+6.56	0.01	+1.65	NS	+8.72	0.0075
**L-ornithine**	-29.00	0.0016	-18.00	0.036	-15.14	NS
**Methylthioadenosine**	-73.68	2.82e-05	-28.00	NS	+63.41	0.001
**N1 or N8-acetyl-spermidine**	-14.00	0.006	-7.80	NS	+26.76	0.001
**N1,N12-diacetlyspermine**	+10.25	1.76e-06	+10.74	1.28e-06	N/A	NS
**N-acetylputrescine**	+36.96	0.0001	+17.59	0.013	-13.96	NS
**Putrescine**	+13.91	0.001	+2.10	NS	+10.36	0.02
**Spermidine**	-6.50	0.011	+3.22	NS	+10.43	0.0009
**Spermine**	+0.77	NS	+21.72	0.004	+20.79	0.02

## Discussion

This study developed and optimised HRMS metabolomics analysis for human plasma and assessed its ability to differentiate stable MCI subjects from MCI subjects who later converted to AD. The robust statistical models generated here indicate the future potential of profiling by HRMS to identify MCI patients at greatest risk of converting to AD. Our findings from pathway enrichment analysis complement and add to prior reports of disturbances in cholesterol, lipid, glucose, amino acid and prostaglandin metabolism in MCI and AD. It also provides new biochemical insights relating to *polyamine* and *L-arginine* metabolism.

The untargeted approach correctly classified subjects with high specificity and sensitivity. Multivariate models predicted cases of MCI with 97.6% accuracy and cases of AD with accuracies of 98.4% (from controls) and 98.7% (from MCI). Within this field few metabolomics-based models have achieved this level of predictive accuracy. Comparable accuracies (>90%) have been achieved with human CSF or brain analysis [9,11,19,25] but studies analysing blood plasma/serum either do not report multivariate models[23] or have had disappointing predictive accuracies (~50%) [24].

The high predictive power of earlier CSF-based studies [9,11] is encouraging and leads to optimism that novel metabolite-based CSF diagnostics can be developed (e.g. biomarkers or disease signatures). The current study indicates that similar diagnostics based on blood plasma are also a possibility. Any improvements to diagnosis or in identifying MCI patients at risk of converting to AD would be significant progress, but if a less invasive approach (i.e. phlebotomy versus lumbar puncture) could be employed then a more universal study of the risk of the population becomes possible in the future. There are still relatively few studies that have applied metabolomics to biofluids from MCI and AD subjects [10,11,23–25] and there is a need to establish disease specific signatures that are reproducible across a number of populations. In this regard we have identified some areas of human metabolism linked to MCI/AD.

Our HRMS metabolomics data performed extremely satisfactorily when interrogated by metabolite pathway enrichment analysis. Only those included ions with assigned identities and significantly between 2 or more groups were included. Stringent criteria (both p-value and FDR lower than 0.05) were applied to identify the most profoundly affected pathways. This implicated just 22 canonical biochemical pathways. Only 7 pathways were affected in stable MCI patients compared with controls, perhaps reflecting the more subtle cognitive changes observed in this condition. It was not entirely unsurprising that a greater number of biochemical pathways were impacted in AD converters: 15 compared with controls, and 14 compared with MCI.

Potentially the most exciting finding was that *polyamine metabolism* and *L-arginine metabolism* were the only two pathways impacted across all group comparisons. Two things were immediately notable, firstly, that in all cases *polyamine metabolism* was more significantly impacted than *L-arginine metabolism;* and secondly, that the two pathways are actually inter-linked sharing common metabolite intermediates. The pathways are directly connected via the urea cycle through the enzyme arginase which converts L-arginine to urea and L-ornithine [26]. Furthermore, arginase appears to control the proliferation of neural cells by modulating the number which enter the S-phase of the cell cycle [27]. Also, the survival of neuronal cells is increased and apoptosis reduced upon administration of arginase [28]. The neural cell benefits of arginase appear to be dependent on the depletion of L-arginine and the inhibition of ‘death protein’ synthesis [29]. However, arginase also appears to exert its effects downstream through the production of various polyamine molecules (i.e. putrescine, spermidine, spermine and their acetylated derivatives) which influences the growth and death of neural cells [30]. Our results indicate that in both stable MCI and in AD converters there is a significant increase in L-arginine which is coupled with a decrease in L-ornithine. This disturbance in the arginase pathway may potentially reflect changes in neurogenesis. Excitingly though, we have observed that *Polyamine metabolism* is differentially affected in stable MCI subjects and AD converting subjects which provides a new insight. Our results suggest that in subjects converting to AD putrescine is channelled towards the production of spermidines and spermines, whereas in stable MCI putrescine has other fates (production of N-acetylputrescine or 4-aminobutanal). Downstream from this there is a major decline in the production of the neurotransmitter γ-aminobutyric acid (GABA) in stable MCI subjects but this does not deteriorate further in AD converting subjects, indicating that GABA decline could be an early contributor to loss in cognitive function. In stable MCI GABA decline is associated with the build-up of GABA precursor metabolites (N-acetylputrescine and 4-aminobutanal) indicating that disruption/blockage of the pathway might occur here. These precursor metabolites do not accumulate in AD converters most likely because putrescine is preferentially metabolised to other polyamines.

The above findings are favourably supported by evidence from the literature. Firstly, brain tissue from the frontal and occipital lobes of AD sufferers has significantly higher levels of purescine, spermine and spermidine [31]. Secondly, elevated levels of Aβ peptides up-regulate polyamine metabolism, increasing polyamine up-take and ornithine decarboxylase (ODC) activity [32], and this appears to be due to elevated neurotoxicity [33]. Thirdly, the expression levels of antizyme inhibitor proteins (AZIN’s) (which increase ODC activity and polyamine concentrations) are elevated in AD [34]. Fourthly, the levels of methythioadenosine (the by-product of spermidine and spermine production) are significantly raised in the CSF of MCI and MCI-AD subjects [11]–although in the present we found methythioadenosine to be differentially affected in the plasma of MCI and MCI-AD subjects. Finally, significant reductions in GABA in AD patients have been reported in the temporal cortex [35] and AD transgenic mice have lower GABA levels in the hippocampus and cortex [36,37]. It should be noted however that reduced levels of GABA in AD are not a universal finding and this remains controversial [10,38].

Apart from *polyamine and L-arginine metabolism* several other areas of metabolism were affected in AD converters, and within the context of AD these appear to be both plausible and relevant. Changes in cholesterol metabolism, glucose metabolism and prostaglandin metabolism were indicated. Pathways involved in cholesterol metabolism: *cholesterol and sphingolipid transport*, *regulation of lipid metabolism* and *transport of intracellular cholesterol* were exclusively impacted in AD converting subjects. There is increasing epidemiological and molecular evidence indicating that cholesterol plays a role in the initiation and/or progression of AD [39,40]. Evidence suggests that disturbed cholesterol metabolism and hypercholesterolemia are important factors in amyloid plaque formation and tau hyperphosphorylation [39]. Also notable from pathway enrichment analysis were alterations in *glycolysis and gluconeogenesis* pathways. In recent years there have been a growing number of studies associating AD with type 2 diabetes [41,42]. In fact, a recent study of 2067 individuals indicated that glucose levels could be a risk factor for dementia even in persons without diabetes [43]. Several potential mechanisms have been suggested including acute and chronic hyperglycemia, insulin resistance and microvascular disease within the central nervous system. Altered *prostaglandin biosynthesis* suggests an underlying inflammatory response in subjects who later convert to AD. The cylcoxygenase (COX) enzymes involved in prostaglandin synthesis are the primary target of nonsteroidal anti-inflammatory drugs. It has been surmised that long term use of these drugs may reduce risk of AD onset [44]. The relationship appears to be complex but randomized controlled prevention trial indicate that rate of cognitive decline is reduced in some cases [45]. This may be result of curbed COX-2 activity, which is normally increased during inflammation producing more of the proinflammatory prostanoids. Furthermore, APP transgenic mice over-expressing of COX-2 display increased amyloid beta plaque formation and greater cognitive deficits [46,47]. Aspects of amino acid metabolism were also affected across subject groups. These include *L-arginine metabolism* (already mentioned), but also *lysine metabolism*, *tryptophan metabolism*, and *tyrosine metabolism*. Disturbed *lysine metabolism* has been previously reported in both MCI and AD subjects [24], but we found it to be only disturbed in stable MCI subjects. Altered *Tryptophan metabolism and tyrosine metabolism* could reflect the importance of these amino acids’ as neurotransmitters i.e. serotonin and noradrenaline/dopamine, respectively.

## Conclusion

This study has developed highly predictive models capable of distinguishing MCI subjects converting to AD up to two years before formal clinical diagnosis. The predictive power of the models is greater than any blood-based metabolomics approach developed thus far. The use of bioinformatics tools to high quality HRMS metabolomics data provides unbiased biologically relevant and meaningful results which are supported by the scientific literature. This investigation has highlighted specific changes in polyamine and L-arginine metabolism which are associated with conversion to AD. There is a need to uncover how these metabolic changes develop and whether this area of metabolism could be the targeted of new AD therapeutics. HRMS metabolomics clearly shows potential for the development of new diagnostic tools for improving patient diagnosis which in the future could allow more stratified/personalised treatment approaches at the memory clinic.

Supplementary information is available at *PLOS ONE’s* website

## Supporting Information

S1 Fig Common and uniquely affected biochemical pathways. Venn diagram illustrates numbers of common and uniquely affected pathways in plasma of Controls, MCI and MCI_AD patients. Just 2 pathways were commonly implicated across all group comparisons: polyamine metabolism and L-arginine metabolism (indicated by white bars in Fig. 3). CHOL- cholesterol, GABA- gamma aminobutyric acid, GNG- gluconeogenesis, HDL- high density lipoprotein, IC- intracellular, NAE- N-acylethanolamine, PG- prostaglandin, PL-phospholipase, UMP- uridine 5’-monophosphate.
(TIF)

S1 Table Characteristics of MCI and Control Participants. (PDF)

S2 Table Classification of MCI Participants According to the Methods of Petersen et al. (1999). (PDF)

S1 Dataset TransOmics Identification Output file. (CSV)

S2 Dataset Raw normalised mass spectral data used to produce multivariate models. (XLSX)
