# Supplementary figures and images for: Untargeted Metabolomic Analysis of Human Plasma Indicates Differentially Affected Polyamine and L-Arginine Metabolism in Mild Cognitive Impairment Subjects Converting to Alzheimer’s Disease
Source: PLoS One. 2015 Mar 24;10(3):e0119452. doi: 10.1371/journal.pone.0119452 (PMC4372431; doi:10.1371/journal.pone.0119452)

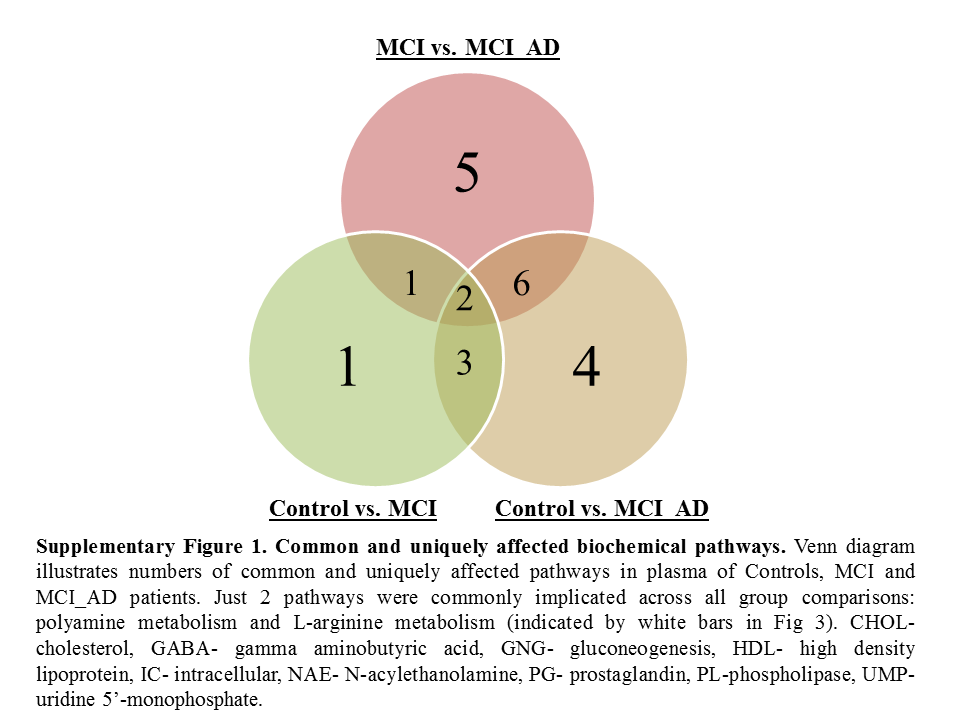

Supplement: S1 Fig — Venn diagram illustrates numbers of common and uniquely affected pathways in plasma of Controls, MCI and MCI_AD patients. Just 2 pathways were commonly implicated across all group comparisons: polyamine metabolism and L-arginine metabolism (indicated by white bars in Fig. 3). CHOL- cholesterol, GABA- gamma aminobutyric acid, GNG- gluconeogenesis, HDL- high density lipoprotein, IC- intracellular, NAE- N-acylethanolamine, PG- prostaglandin, PL-phospholipase, UMP- uridine 5’-monophosphate. (TIF) [file pone.0119452.s001.tif]
