# Supplementary material for: Untargeted Metabolomic Analysis of Human Plasma Indicates Differentially Affected Polyamine and L-Arginine Metabolism in Mild Cognitive Impairment Subjects Converting to Alzheimer’s Disease
Source: PLoS One. 2015 Mar 24;10(3):e0119452. doi: 10.1371/journal.pone.0119452 (PMC4372431; doi:10.1371/journal.pone.0119452)
Supplement: S1 Table — (PDF) [file pone.0119452.s002.pdf]

Supplementary Table 1. Characteristics of MCI and Control Participants.

	**Control (n=37)**	**MCI (n=16)**	**MCI_AD (n=19)**	**F df p**
**Age (years: mean (sd))**<br><br>**Range (min-max)**	73.1 (8.9)<br><br>56-92	72.4 (7.3)<br><br>56-80	77.9 (4.4)<br><br>67-84	5.00 2 **0.03**
**NART IQ (Mean (sd))**	112.5 (8.7)	112.7 (7.1)	115.1 (7.3)	0.73 2 0.40
**Years of education(Mean (sd))**	11.8 (2.9)	12.0 (3.1)	11.9 (2.3)	0.01 2 0.91
**MMSE (Mean (sd))**	29.4 (0.8)	28.5 (1.3)	27.1 (1.4)	15.3 2 **<0.01**
**ACE (Mean (sd))**	92.0 (2.4)	86.8 (4.3)	81.8 (7.8)	9.34 2 <**0.01**
**DAD (Mean (sd))**	80.0 (0.1)	79.7 (0.8)	79.7 (0.7)	0.09 2 0.77
**Gender F:M**	19:18	8:8	12:7	χ^2=0.85 2 0.65

*ANOVA Analysis; National Adult Reading Test [NART]; Mini Mental State Examination [MMSE]; Disability Assessment in Dementia [DAD]; Addenbrookes Cognitive Examination [ACE].
