# Supplementary material for: Untargeted Metabolomic Analysis of Human Plasma Indicates Differentially Affected Polyamine and L-Arginine Metabolism in Mild Cognitive Impairment Subjects Converting to Alzheimer’s Disease
Source: PLoS One. 2015 Mar 24;10(3):e0119452. doi: 10.1371/journal.pone.0119452 (PMC4372431; doi:10.1371/journal.pone.0119452)
Supplement: S2 Table — (PDF) [file pone.0119452.s003.pdf]

Supplementary Table 2: Classification of MCI Participants According to the Methods of Petersen et al. (1999).

Group Categorisation	MCI patients (n=16)	MCI_AD patients (n=19)
**Amnestic single domain**	4	1
**Amnestic multidomain**	6	16
**Nonamnestic single domain**	5	1
**Nonamnestic multidomain**	1	1
